# Development and evaluation of recombinase-aided amplification assays incorporating competitive internal controls for detection of human adenovirus serotypes 3 and 7

**DOI:** 10.1186/s12985-019-1178-9

**Published:** 2019-07-01

**Authors:** Rui-huan Wang, Hong Zhang, Yi Zhang, Xin-na Li, Xin-xin Shen, Ju-ju Qi, Guo-hao Fan, Xing-yu Xiang, Zhi-fei Zhan, Zi-wei Chen, Xue-jun Ma

**Affiliations:** 1 University of South China, College of Public Health, No. 28, West Changsheng Street, Hengyang, 421001 Hunan China; 2 Hunan Provincial Center for Disease Control and Prevention, No. 450, Furong Street, Changsha, 410005 Hunan China; 3 NHC Key Laboratory of Medical Virology and Viral Diseases, National Institute for Viral Disease Control and Prevention, Chinese Center for Disease Control and Prevention, No. 155, Changbai Street, Changping District, Beijing, 102206 China; 4 The Third Xiangya Hospital of Central South University, 138 Tongzipo Road, Yuelu District, Changsha City, 410013 Hunan Province China

**Keywords:** Pneumonia, Human adenovirus, Duplex recombinase aided amplification, Detection

## Abstract

**Background:**

Human adenoviruses are a common group of viruses that cause acute infectious diseases. Human adenovirus (HAdV) 3 and HAdV 7 cause major outbreaks of severe pneumonia. A reliable and practical method for HAdV typing in clinical laboratories is lacking. A simple, rapid and accurate molecular typing method for HAdV may facilitate clinical diagnosis and epidemiological control.

**Methods:**

We developed and evaluated duplex real-time recombinase-aided amplification (RAA) assays incorporating competitive internal controls for detection of HAdV 3 and HAdV 7, respectively. The assays were performed in a one-step in a single tube reaction at 39° for 20 min.

**Results:**

The analytical sensitivities of the duplex RAA assays for HAdV 3 and HAdV 7 were 5.0 and 14.8 copies per reaction, respectively (at 95% probability by probit regression analysis). No cross-reaction was observed with other types of HAdV or other common respiratory viruses. The duplex RAA assays were used to detect 152 previously-defined HAdV-positive samples. These results agreed with those obtained using a published triplex quantitative real-time PCR protocol.

**Conclusions:**

We provide the first report of internally-controlled duplex RAA assays for the detection of HAdV 3 and HAdV 7. These assays effectively reduce the rate of false negative results and may be valuable for detection of HAdV 3 and HAdV 7 in clinical laboratories, especially in resource-poor settings.

## Background

Human adenoviruses are non-enveloped, spherical, double-stranded DNA viruses belonging to the *Adenoviridae* family and the *Mastadenovirus* genus. Human adenovirus (HAdV) comprises of seven species (A–G), and > 90 serotypes have been identified based on biophysical, biochemical and genetic characteristics (http://hadvwg.gmu.edu/). HAdV is commonly detected in the pediatric population. Different serotypes of HAdV result in distinct clinical manifestations, including acute respiratory infections, conjunctivitis, pneumonia, gastroenteritis, myocarditis, and even central nervous system dysfunction [1, 2]. Previous studies reported that HAdV 3 and HAdV 7 were the main genotypes causing outbreaks of infection in many countries [3], including Singapore, the United States, the United Kingdom, Korea [4] and Canada [2, 5]. In China, major outbreaks have happened in Taiwan [6], Chongqing [7] and Nanjing [8]. Deng Jie et al. [9] investigated acute respiratory infections among pediatric patients in Beijing, with HAdV 3 and HAdV 7 accounting for > 90% of infections. HAdV 3 and HAdV 7 were also the main causative pathogens of lower respiratory tract infection epidemics that caused fatalities [10, 11]. Therefore, it is critical to develop sensitive and timely detection methods for HAdV 3 and HAdV 7, which may facilitate clinical diagnosis and epidemiological studies.

Traditional detection methods for HAdV 3 and HAdV 7, such as immunoassays and viral culture [12], are not suitable for rapid disease diagnosis because they are time-consuming and laborious. Commonly used molecular diagnostic assays for the identificition HAdV 3 and HAdV 7 with high sensitivity and specificity, such as temperature switched amplification based on polymerase chain reaction (PCR) [13], fluorescence based real-time PCR [14, 15], and nested PCR [16], require a thermal cycler and well trained specialists, which is not practical for rapid and convenient detection in low income areas. Compared with traditional methods, isothermal nucleic acid amplifications methods such as loop mediated isothermal amplification (LAMP) [17] and nucleic acid sequence based amplification [18] are simple, rapid and cost-effective.

Here, we present a novel, isothermal, recombinase-aided amplification (RAA) assay which does not require a classical thermostable enzyme or a sophisticated thermal cycler. The enzymes used in the RAA assay include single strand DNA binding protein (SSB), recombinase UvsX, and DNA polymerase. The reaction is typically completed at 39 °C in 20–30 min. RAA has been successfully applied in the detection of bacterial and viral pathogens [19–21]. However, none of these methods included internal controls (ICs).An IC may be a competitive IC or a non-competitive IC. For a non-competitive IC, a set of primers is used for targeting the IC template, and a further set of primers is used for targeting the test template. However, different primers might have amplification bias and reduce the amplification efficiency of the target template. For a competitive IC, only one pair of primers is used in amplification, ensuring consistency of amplification. Occasionally, with an extremely high concentration of sample, amplification of the IC may be inhibited by competitive amplification. However, in such cases detection results are considered positive since the IC is only used to prevent false negative results [22]. The simultaneous amplification of two different DNA fragments from the same primer set may inhibit or enhance one or both of the products, depending on the molar ratio, length, sequence and secondary structure of the DNA fragments [23]. Therefore, the concentration of the IC used is important. The present study aimed to integrate RAA using fluorescence detection. We designed specific primers and probes and added a competitive IC as a quality control and thus established RAA assays for detection of HAdV 3 and HAdV 7 at 39 °C in 20 min. Additionally, we tested clinical samples and compared the performance of the RAA with a published triplex quantitative real-time PCR (tq-PCR) protocol [15].

## Results

### Singleplex RAA assay sensitivity and limit of detection

The sensitivity of singleplex RAA assays for HAdV 3 and HAdV 7 (i.e. containing samples but no IC) was determined using diluted recombinant standard plasmids. The detection limits of the singleplex RAA assays for HAdV 3 and HAdV 7 at 95% probability (probit analysis) were 4.4 and 5.0 copies/reaction, respectively (Table 1). No signal was detected for any of the negative controls.

**Table 1:** Detection limits of HAdV 3 and HAdV 7 in singleplex and duplex RAA assays

Copies/reaction	No. of positive samples/no. of samples tested by the RAA assay for detection of HAdV 3 and HAdV 7			
	Singleplex RAA		Duplex RAA	
	HAdV 3	HAdV 7	HAdV 3	HAdV 7
1 copy/μL	6/8	2/8	2/8	0/8
10^1^ copy/μL	8/8	8/8	8/8	4/8
10^2^ copy/μL	8/8	8/8	8/8	8/8
10^3^ copy/μL	8/8	8/8	8/8	8/8
10^4^ copy/μL	8/8	8/8	8/8	8/8

### Duplex RAA assay sensitivity and specificity

**Fig. 1:**
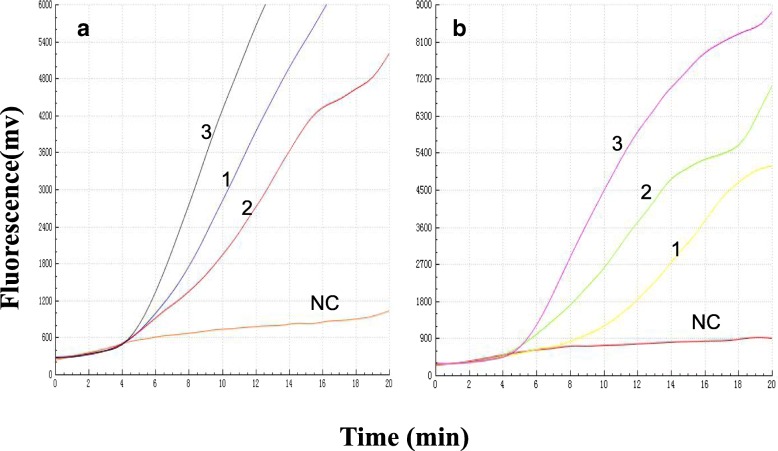
**a** Amplification curves 1 to 3 indicate a dilution range from 10^1^ to 10^3^ copies/reaction of the internal control (IC) with 100 copies of HAdV 3 standard plasmid. **b** Amplification curves 1 to 3 indicate a dilution range from 10^1^ to 10^3^ copies/reaction of the IC with 100 copies of HAdV 7 standard plasmid

**Fig. 2:**
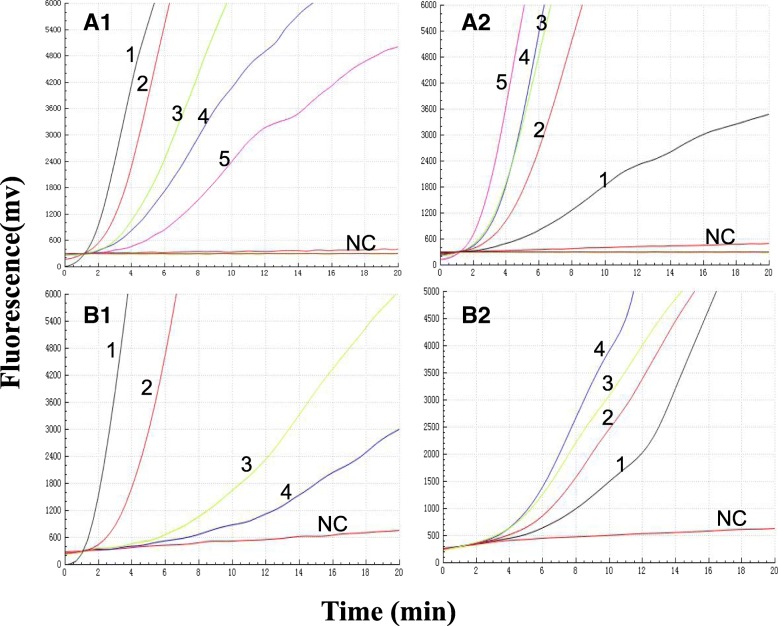
A1: Amplification curves 1 to 5 indicate a dilution range from 10^4^ to 10^0^ copies/reaction of HAdV 3 standard plasmid with 1000 copies of the IC in the HEX (5-hexachlorofuorescein) detection channel. **A2**: Amplification curves for 1000 copies of the IC combined with (curves 1 to 5) 10^4^ to 10^0^ copies/reaction of HAdV 3 standard plasmid, in the FAM (6-carboxyfuorescein) detection channel. The same color amplification curves in **A1** and **A2** indicate use of the same pair of primers in the reaction tube. B1: Amplification curves 1 to 4 indicate a dilution range from 10^4^ to 10 copies/reaction of HAdV 7 standard plasmid with 1000 copies of the IC in the HEX detection channel. **B2**: Amplification curves for 1000 copies of the IC combined with (curves 1 to 4) 10^4^ to 10 copies/reaction of HAdV 7 standard plasmid, in the FAM detection channel. The same color amplification curves in **B1** and **B2** indicate use of the same pair of primers in the reaction tube

The optimum concentration of the IC template was determined to be 1 × 10^3^ copies in each internally-controlled duplex RAA assay. This concentration did not inhibit amplification of 100 copies of the target gene (Fig. 1). The detection limits of the duplex RAA assays for HAdV 3 and HAdV 7 were 5.0 and 14.8 copies/reaction, respectively (Fig. 2; Table 1). As shown in Fig. 2, the higher the concentration of the virus target plasmid, the earlier the peak was observed, and the later the peak corresponding to the IC was observed. As shown in Table 1, results indicated that both HAdV 3 and HAdV 7 duplex RAA assays were capable of detecting 50 copies of the respective virus target per reaction.

A total of 64 other samples positive for common respiratory infections were respectively detected using the duplex RAA assays to test the specificity of the method. These pathogens included influenza A and B viruses, rhinovirus, parainfluenza virus, human bocavirus, coronavirus, human metapneumovirus, respiratory syncytial virus, and other HAdV species—A (HAdV 31), B (HAdV 14, 55), C (HAdV 1, 2, 5, 6, 57), and E (HAdV 4). No fluorescence signal was detected from any of these samples, indicating that the duplex RAA assays showed no cross-reactions with these pathogens (data not shown).

### Clinical evaluation of duplex RAA assays and agreement between duplex RAA assays and tq-PCR

**Fig. 3:**
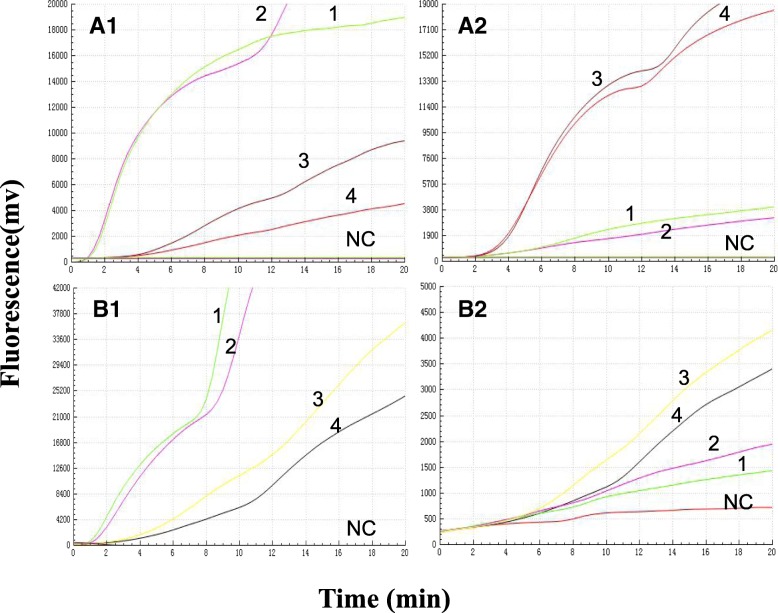
**A1** Amplification curves 1 to 4 indicate HAdV 3 clinical samples with triplex quantitative real-time PCR (tq-PCR) cycle threshold (CT) values of 16.48, 20.39, 36.42, and 36.45 with 1000 copies of the IC in the HEX detection channel. **A2** Amplification curves for 1000 copies of the IC combined with (curves 1 to 4) samples as in **A1**, in the FAM detection channel. The same color amplification curves in **A1** and **A2** indicate use of the same pair of primers in the reaction tube. **B1** Amplification curves 1 to 4 indicate HAdV 7 clinical samples with tq-PCR CT values of 15.85, 17.65, 33.57, and 35.99 with 1000 copies of the IC in the HEX detection channel. **B2** Amplification curves for 1000 copies of the IC combined with (curves 1 to 4) samples as in **B1**, in the FAM detection channel. The same color amplification curves in **B1** and **B2** indicate use of the same pair of primers in the reaction tube

To evaluate the performance of the duplex RAA assays in the detection of HAdV 3 and HAdV 7, a total of 152 HAdV-positive clinical samples were detected with the duplex RAA assays. Tq-PCR results showed that there were 43 HAdV 3 samples and 58 HAdV 7 samples. A threshold cycle (CT) value in tq-PCR < 38 was considered a positive result. As shown in Fig. 3, the CT values of the HAdV 3 clinical samples ranged from 16.48 to 36.45, and the CT values of the HAdV 7 samples ranged from 15.85 to 35.99. The duplex RAA assays were able to detect weakly-positive specimens in tq-PCR, with CT values > 35. Compared with tq-PCR (Table 2), the kappa values of the duplex RAA assays for HAdV 3 and HAdV 7 were 1.0 (*p* < 0.001) and 1.0 (p < 0.001), respectively. That is, the results of tq-PCR and the duplex RAA assays were fully in agreement. However, the duplex RAA takes only 20 min, while tq-PCR requires complex equipment and takes at least an hour.

**Table 2:** Detection of HAdV 3 and HAdV 7 in clinical samples

Virus	Duplex RAA		tq-PCR		Agreement
	Positive	Negative	Positive	Negative	
HAdV 3	43	0	43	0	100%
HAdV 7	58	0	58	0	100%

## Discussion

HAdV 3 and HAdV 7 are serotypes responsible for worldwide severe HAdV infections [2, 12, 13], and may cause successive outbreaks of acute respiratory or central nervous system infection [4, 6, 24]. In China, epidemic outbreaks of HAdV 3 and HAdV 7 have been reported in Shanghai [25], Taiwan [6, 26], Nanjing [8], Beijing [9], and Chongqing [7]. Thus, a simple, rapid and accurate classification of HAdV 3 and HAdV 7 in HAdV detection is particularly desirable.

In this study, we first reported singleplex RAA assays for HAdV 3 and HAdV 7, respectively. Detection limits of these methods were 4.4 and 5.0 copies per reaction, respectively. These assays were further developed by introducing an IC to create two duplex real-time fluorescent RAA assays. The introduction of the IC effectively eliminated false negative results and invalid results.

Isothermal nucleic acid amplification assays including LAMP and RAA are increasingly used in virus detection [17, 19, 21, 27], but few studies have reported the use of an IC in isothermal assays. ICs are required to distinguish between true negative results and reactions that simply failed. Detection is considered negative when there is amplification of the IC only. If neither the IC nor the sample is amplified, an invalid assay is indicated [22]. As LAMP requires multiple primers, potential interaction between primers may lead to a difficult and complex situation for introducing an IC [28]. The RAA primer and probe are > 30 bp in length, and long nucleic acid sequences easily form secondary structure, which is the major obstacle to developing a duplex RAA method including an IC.

**Fig. 4:**
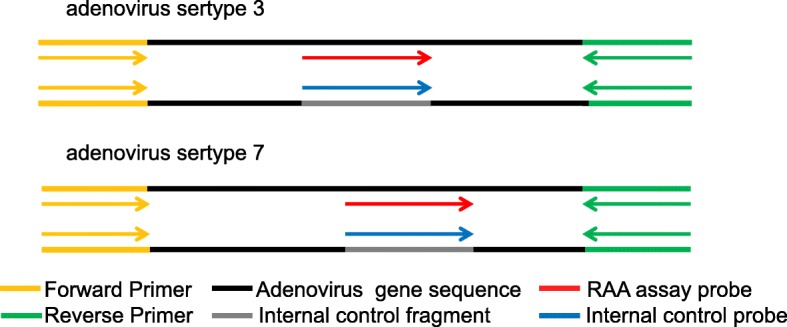
Schematic diagram of duplex recombinase-aided amplification (RAA) assays for detection of HAdV 3 and HAdV 7

In the present study, we adopted a competitive IC strategy with one pair of common primers in the same reaction tube. The only sequence difference between the target sequence and the IC sequence was in the probe binding region (Fig. 4). Using 100 copies of the target gene, we determined the optimum concentration of the IC as 1 × 10^3^ copies. By optimizing the concentration of the IC template, we successfully established duplex RAA assays incorporating a competitive IC to avoid false negative results.

The duplex RAA detection limits of HAdV 3 and HAdV 7 were 5.0 and 14.8 copies per reaction (probit analysis, *p* ≤ 0.05). Further application of the two methods to 48 HAdV 3-positive and 53 HAdV 7-positive clinical samples (identified by tq-PCR) showed 100% consistency of results. Seven samples that were weakly positive by tq-PCR (CT values > 35) were identified as positive by the duplex RAA. In addition, no cross-reaction was observed with other common respiratory viruses or other HAdV species [A (HAdV 31), B (HAdV 14, 55), C (HAdV 1, 2, 5, 6, 57) or E (HAdV 4)] in clinical samples, indicating high sensitivity and specificity of the RAA.

Compared with traditional PCR, the duplex RAA assay is convenient and fast (20 min at 39 °C) without the need for complicated operations or expensive variable-temperature PCR instrument. This assay was performed in a single closed tube, leading to a contamination-free test. The clinical samples tested with this assay were from two cities, one in southern China, the other in northern China, suggesting the adaptability of the duplex RAA assays for HAdV 3 and HAdV 7 detection.

More comprehensive evaluation of the duplex RAA assays using larger sets of clinical samples will be needed in the future. We will also attempt to combine the RAA with a lateral flow dipstick test for visual detection to avoid the need for a fluorescence detection device, and explore direct RAA detection of HAdV 3 and HAdV 7 by adding lytic solution to the original sample without nucleic acid extraction.

## Conclusions

RAA assays for the detection of HAdV 3 and HAdV 7 with high sensitivity and specificity were demonstrated here. The method is rapid and does not require a thermal cycler. By introducing an IC, the duplex RAA assays successfully eliminated false negative results, thereby producing much more reliable tests. This method might have great potential for clinical use, especially in resource-poor settings.

## Materials and methods

### Sample information

A total of 256 clinical samples from patients with febrile respiratory syndrome were collected at the Hunan Provincial Center for Disease Control and Prevention and the Children’s Hospital of Hebei Province (China) from 2014 to 2017, including 196 nasopharyngeal aspirates and 60 throat swabs. They were stored at − 80 °C until extraction of nucleic acids. These specimens had previously been tested in a GeXP-based multiplex RT-PCR assay [29] for common respiratory viruses and 152 were found to be positive for HAdV. This study was conducted with the approval of the Institutional Review Boards of the National Institute for Viral Disease Control and Prevention, Center for Disease Control and Prevention of China. Written informed consent was obtained from children’s caregivers.

### Nucleic acid extraction

Total DNA was extracted from 200 μL of each clinical sample using a Viral RNA/DNA Isolation Kit (Tianlong, Suzhou, China) according to the manufacturer’s protocol. The DNA was eluted in 50 μL of DNase- and RNase-free water and stored at − 80 °C until use.

### Preparation of recombinant plasmids

A 270-bp (nt 18,754–19,023, GenBank accession no. EF564601.1) and a 299-bp fragment (nt 18,486–18,784, GenBank accession no. KX897164.1) of the hexon genes of HAdV 3 and HAdV 7 were respectively cloned into vector pUC57 for DNA copy number quantification. The plasmid DNA was quantified using a Qubit 2.0 fluorometer (Life Technologies, Warrington, UK). The resulting DNA concentrations were converted to genome copy values using the formula:

DNA copy number (copy number/μL) = [6.02 × 10^14^ × plasmid concentration (ng/μL) × 10^− 9^]/[DNA length in nucleotides × 660].

The plasmids were diluted 10-fold from 10^8^ to 10^0^ copies /μL, and stored at − 20 °C.

### Design of primers and probes

Eighty complete hexon gene sequences of HAdV 3 and HAdV 7 were downloaded from NCBI GenBank, and the gene sequences were aligned using VectorNTI. Then the forward primer, reverse primer and probe were designed using Oligo7 according to the principle of minimizing secondary structure. Two RAA probes were also designed to target the IC templates for use in HAdV 3 and HAdV 7 duplex RAA assays. The IC template was a short gene sequence of rose rosette virus, which was inserted into the plasmids encoding HAdV 3 and HAdV 7 replacing the probe sequences of HAdV 3 and HAdV 7, respectively (Fig. 4). The cloning was conducted by TsingKe Biotech Corp. (Beijing, China). The HAdV probes and IC probes were labelled with 5-hexachlorofuorescein (HEX) and 6-carboxyfuorescein (FAM) fluorophores, respectively. All the primer and probe sequences are listed in Table 3. The primers and probes were synthesized by Shanghai Bioengineering (Shanghai, China).

**Table 3:** Primers and probes used for RAA and tq-PCR

	Primer/probe	Sequence (5′ to 3′)	Product size (bp)	Source
RAA	AdV 3-F	ATTCCGGCACAGCTTACAATTCACTCGCTCC	184	This paper
	AdV 3-R	TCAGTAGTGG TAATGTCTTT CCCAATTTGC		
	AdV 3-P^a^	ACAATGCAGTAACTACCACCACAAACACA [HEXdT][THF][BHQ-dT]GGCATTGCTTCCAT[C3-spacer]		
	AdV 7-F	ACAACGGGAGAAGACAATGCCACCACATACAC	186	Thispaper
	AdV 7-R AdV 7-P^a^ IC-P^a^	TCCATCAATATCAGTCCATGATTCTTCTCC AAGACATTACTGCAGACAACAAGCCCATT [HEXdT][THF][BHQ-dT]GCCGATAAAACATAT[C3-spacer] GTAAGGTGCTAGACTAAAATTGTTGGGACTT [FAMdT]G[THF]A[BHQ-dT]CTCTGAAGTAAAAGG[C3-spacer]		
tq-PCR	F-primer	GGYCCYAGYTTYAARCCCTAYTC		[15]
	R-primer	AAYTTGAGGYTCTGGYTGATCKG		
	Probe3	HEX-ACAATGCAGTAACTACCACCACAA-MGB		
	Probe7	Cy5-TTACTGCAGACAACAAGCCCAT-MGB		

^a^Probe modifications: *FAM* 6-carboxyfuorescein, *HEX* 5-hexachlorofuorescein, *THF* Tetrahydrofuran, *BHQ* Black hole quencher, *C3-spacer* 3′ phosphate blocker

### Singleplex RAA assay protocol, analytical sensitivity and limit of detection

The singleplex real time RAA assay was performed in a 50-μl reaction volume using the RAA exo kit (Jiangsu Qitian Bio-Tech Co. Ltd., China). The reaction components included 25 μL rehydration buffer, 16.7 μL DNase-free water, 2.1 μL of 10 μM forward and reverse primers, 0.6 μL of 10 μM target specific RAA exo-probe, and 1 μL DNA template or 1 μL DNase-free water. The 47.5-μL reaction mixture was added to the lyophilized RAA pellet kit which contains all necessary enzymes (SSB, 800 ng/μL; UvsX, 120 ng/μL; DNA polymerase, 30 ng/μL), then 2.5 μL of 280 mM magnesium acetate were pipetted into the caps. The tube lids were carefully sealed and the tubes were shaken and centrifuged by vortex instrument for 4 min. Then, the tubes were transferred to the tube holder on the QT-RAA-F1620 real-time fluorescence detection system (Jiangsu Qitian Bio-Tech Co. Ltd.) at 39 °C for 20 min. The RAA assay sensitivity for detection of HAdV 3 and HAdV 7 was determined using 10-fold dilutions of recombinant standard plasmid (10^4^ to 10^0^ copies per reaction, *n* = 8). Negative control reactions were performed in parallel in each run.

### Development of internally-controlled duplex RAA assays

The HAdV 3 and HAdV 7 singleplex RAA assays were further developed to establish internally-controlled duplex RAA assays by incorporating an IC template and corresponding exo probe. The real-time RAA reaction was performed in a 50-μL volume using the exo kit (Jiangsu Qitian Bio-Tech Co. Ltd.): 25 μL rehydration buffer, 15.1 μL DNase-free water, 2.1 μL of forward and reverse primers (10 μM), 0.6 μL of target specific exo probe (10 μM), 0.6 μL of IC exo probe (10 μM), 1 μL IC template, 1 μL DNA template or 1 μL DNase-free water, and 2.5 μL of 280 mM magnesium acetate. The specific reaction steps were performed as described for the singleplex RAA reactions. The optimum concentration of the IC template was evaluated by testing 100 copies of target plasmids in the presence of various IC copy concentrations (1 × 10, 1 × 10^2^, 1 × 10^3^). Standard recombinant plasmids with a dilution range 10^4^–0^0^ were prepared to determine the sensitivity of duplex RAA assays for detection of HAdV 3 and HAdV 7, respectively. The method of determination of sensitivity and the limit of detection was as described above. Specificity was evaluated by retrospective testing of 64 samples positive for other respiratory viruses or other types of HAdV.

### Evaluation of the duplex RAA assays using clinical samples and comparison with tq-PCR

To evaluate the clinical performance, a total of 152 clinical samples previously confirmed HAdV-positive by a GeXP-based multiplex RT-PCR assay were nucleic acid extracted again, then detected in duplicate by the duplex RAA assays. A reference tq-PCR assay for the detection of HAdV 3 and HAdV 7, respectively, was performed in parallel on these samples. A tq-PCR threshold cycle (CT) value of < 38 was determined as a positive result. A slope of the RAA amplification curve > 20 set by the detection device was determined as positive.

## Abbreviations

HAdV: Human adenovirus
IC: Internal control
PCR: Polymerase chain reaction
RAA: Recombinase-aided amplification
tq-PCR: Triplex quantitative real-time PCR
